# Subgenome Bias and Temporal Postponement of Gene Expression Contributes to the Distinctions of Fiber Quality in *Gossypium* Species

**DOI:** 10.3389/fpls.2021.819679

**Published:** 2021-12-23

**Authors:** Huan Mei, Bowen Qi, Zegang Han, Ting Zhao, Menglan Guo, Jin Han, Juncheng Zhang, Xueying Guan, Yan Hu, Tianzhen Zhang, Lei Fang

**Affiliations:** ^1^Department of Agronomy, Institute of Crop Science, College of Agriculture and Biotechnology, Zhejiang University, Hangzhou, China; ^2^Hainan Institute of Zhejiang University, Sanya, China

**Keywords:** allotetraploid cotton, homoeologous expression bias, temporally postponed expression, co-expression network, fiber development

## Abstract

As two cultivated widely allotetraploid cotton species, although *Gossypium hirsutum* and *Gossypium barbadense* evolved from the same ancestor, they differ in fiber quality; the molecular mechanism of that difference should be deeply studied. Here, we performed RNA-seq of fiber samples from four *G. hirsutum* and three *G. barbadense* cultivars to compare their gene expression patterns on multiple dimensions. We found that 15.90–37.96% of differentially expressed genes showed biased expression toward the A or D subgenome. In particular, interspecific biased expression was exhibited by a total of 330 and 486 gene pairs at 10 days post-anthesis (DPA) and 20 DPA, respectively. Moreover, 6791 genes demonstrated temporal differences in expression, including 346 genes predominantly expressed at 10 DPA in *G. hirsutum* (TM-1) but postponed to 20 DPA in *G. barbadense* (Hai7124), and 367 genes predominantly expressed at 20 DPA in TM-1 but postponed to 25 DPA in Hai7124. These postponed genes mainly participated in carbohydrate metabolism, lipid metabolism, plant hormone signal transduction, and starch and sucrose metabolism. In addition, most of the co-expression network and hub genes involved in fiber development showed asymmetric expression between TM-1 and Hai7124, like three hub genes detected at 10 DPA in TM-1 but not until 25 DPA in Hai7124. Our study provides new insights into interspecific expression bias and postponed expression of genes associated with fiber quality, which are mainly tied to asymmetric hub gene network. This work will facilitate further research aimed at understanding the mechanisms underlying cotton fiber improvement.

## Introduction

Cotton (*Gossypium* spp.) is an important economic crop throughout the world. Currently, two types of allotetraploid cotton are widely cultivated, namely *Gossypium hirsutum* and *Gossypium barbadense*. Of these, *G. hirsutum* evidences a wider range of adaptability, high yield potential, and moderate fiber quality, leading to it accounting for about 90% of the world’s cotton productivity, while *G. barbadense* produces exceptionally high-quality, extra-long fibers and so enables special cotton textile manufacturers to be competitive. These allotetraploid cotton species originated from an allomorphic event about 1–2 million years ago (Wang et al., 2017), evolved independently, and were domesticated in different geographic areas (Paterson et al., 2012; Hu et al., 2019). *G. hirsutum* is native to the Mesoamerican and Caribbean regions, while *G. barbadense* is indigenous to the coastal areas of Peru (Westengen et al., 2005). Since their divergence and dual domestication, reciprocal but asymmetric introgression between these species has greatly improved cultivar productivity and fiber quality (Fang et al., 2017, 2021). Continuing to introduce superior fiber traits from *G. barbadense* into *G. hirsutum*, and especially doing so more precisely, remains of considerable interest for cultivating *G. hirsutum* to produce longer, finer, and stronger fibers (Lu et al., 2017; Song et al., 2017).

The fiber cells of cotton are a kind of plant cell that is easy to separate and has a secondary cell wall (SCW) composed primarily of celluloses (more than 90%). As a result, in addition to the great economic value of cotton fiber, these cells provide a unique single-cell model system for studying cell elongation and cell wall biosynthesis (Haigler et al., 2012). Cotton fiber development can be divided on the basis of morphological characteristics into four different but overlapping stages, including fiber initiation [0–3 days post-anthesis (DPA)], elongation (3–20 DPA), SCW biosynthesis (15–40 DPA), and maturation (40–50 DPA). On the day of anthesis, the cotton fiber initiates from the epidermis of the ovule.

In recent years, cotton functional genomics studies have provided new insights into fiber development, including interspecies differences. A few transporters such as *GbTST1*, *GbNHX1*, and *GbALMT16* were identified as having a longer period of expression in Hai7124 than their orthologs in TM-1, which could result in more sucrose, K^+^, and malate being pumped into the vacuole; in addition, Hai7124 expressed more genes associated with membrane transport, transcription, glycan biosynthesis, and carbon metabolism (Hu et al., 2019). The expression bias of homologous genes among polyploid subgenomes is also a subject of intense research. RNA-based gene expression assays comparing tetraploid domesticated species and wild species have revealed artificial domestication caused more fiber development-related genes to be expressed and involved in various basic metabolism pathways such as fatty acid, amino acid, and organic acid metabolism (Yoo et al., 2013; Nomaguchi et al., 2018). In addition, complete whole-genome sequencing and assembly of several allotetraploid cotton species has provided key references for functional genomics research (Li et al., 2015; Zhang et al., 2015; Hu et al., 2019; Wang et al., 2019; Ma et al., 2021). Using these and other data, differences in gene expression and regulation networks can be identified, which is necessary to elucidate the global genetic and molecular bases of interspecies differences in fiber quality.

In this study, we performed transcriptome analysis of four *G. hirsutum* and three *G. barbadense* cultivars to conduct joint research into differences in gene expression and regulation networks, the better to reveal critical differential factors at key fiber development stages, including biased expression, patterns of postponed expression, and different co-expression networks. In particular, we aimed to identify new candidate genes as potential determinants of fiber quality and gain new insights into distinct mechanisms for fiber quality improvement.

## Materials and Methods

### Plant Materials and RNA Generation

Four cultivars of *G. hirsutum* (TM-1, XLZ42, 4005, J220) and three of *G. barbadense* (Hai7124, 3–79, Coastland R4-4) were planted in a standard field condition. RNA was collected from fibers of these cotton plants and subjected to transcriptome sequencing. TM-1 and Hai7124 samples at 0, 1, 3, 5, 10, 20, and 25 DPA were obtained during a previous study in our lab, and the data have been uploaded to the NCBI SRA (PRJNA490626) (Hu et al., 2019). For other cultivars, fiber samples were collected at 10 and 20 DPA, respectively representing the stages of fiber elongation and SCW biosynthesis. The ovules and fibers were dissected immediately after harvesting, frozen in liquid nitrogen, and stored at −80°C until being subjected to transcriptome sequencing. The Illumina HiSeq 4000 sequencing platform was utilized to generate 150-bp paired-end reads. Three biological replicates were performed for most samples.

### Generation and Processing of Data

All bioinformatics analyses were performed in accordance with standard procedures. First, the raw data in FASTQ format was processed *via* fastp version 0.19.7 (Chen et al., 2018) to obtain clean reads, and then the Q20, Q30, and GC content of those reads were calculated. Second, all clean reads were aligned to the reference TM-1 genome (v2.1) or Hai7124 genome (v1.1) (Hu et al., 2019) as appropriate using hisat2 version 2.1.0 (Kim et al., 2015), and quantification of gene expression was performed with featureCounts version 2.0.0 (Liao et al., 2014) using the GTF annotation file. To accurately allocate homologous gene reads counts between two subgroups (At and Dt), we considered the reads with mapping quality >15 as uniquely mapped reads, and the mapping ratio and unique mapping ratio were 93.50 and 83.14% (Supplementary Table 1), respectively. For small portion of multi-mapping read, the StringTie-HISAT2 approach could correct the multi-mapping with the parameter with -u (Pertea et al., 2016). The fragments per kilobase of transcript per million mapped reads (FPKM) was calculated for estimating gene expression levels. Gene Ontology (GO) and Kyoto Encyclopedia of Genes and Genomes (KEGG) pathway^1^ enrichment analyses were performed using TBtools version 1.046 (Chen et al., 2020). Principal component analysis (PCA) (Wold et al., 1987) and determination of Pearson correlation coefficients (Benesty et al., 2009) were conducted with R version 4.0.2.

### Evaluation of Homolog Expression Bias

The biased genes were calculated as the same method in previous research (Zhang et al., 2015; Yang et al., 2016). To evaluate expression bias between 21,419 orthologous gene pairs at each fiber development stage, Fragments Per Kilobase Million (FPKM) values for each gene pair were compared using Fisher’s exact test (Mehta and Patel, 1983). *P*-values were adjusted using the Benjamini-Hochberg method (Thissen et al., 2002). Differential expression between gene pairs was delimited by 2-fold expression changes with a false discovery rate (FDR) < 0.05 (Benjamini and Hochberg, 1995). In terms of relative expression, FPKM (A subgenome) > FPKM (D subgenome) indicated the biased expression of A orthologs while FPKM (A subgenome) < FPKM (D subgenome) indicated the biased expression of D orthologs.

### Identification of Temporal Expression Patterns

The R package *Mfuzz* (Kumar and E Futschik, 2007) was used to analyze temporal expression trends and divide genes into several clusters. All genes expressed by TM-1 and Hai7124 were divided into clusters according to expression level to research overall trends of expression in these two cultivars. Orthologs were then used to compare expression patterns and identify genes with special expression patterns in either cultivar. The postponed genes were homoeologous genes expressed later in Hai7124 compared to those in TM-1.

### Construction of Gene Co-expression Networks and Selection of Hub Genes

Orthologous gene co-expression networks were constructed for TM-1 and Hai7124 *via* the R package weighted gene co-expression network analysis (WGCNA) (Langfelder and Horvath, 2008). Genes that clustered in modules for which the highest coefficient was associated with a specific fiber developmental stage were used for further analysis. The genes in edge files were sorted by weight value, and the first 50∼100 pairs were used to establish gene interaction networks. Hub genes were selected on the basis of module membership (K_*ME*_) values, and the interaction network was visualized using Cytoscape version 3.8.0 (Cline et al., 2007).

### Validation of Genes With Postponed Expression by Quantitative Real-Time Polymerase Chain Reaction

To verify the differential temporal expression patterns observed in TM-1 and Hai7124, three genes were selected for quantitative real-time polymerase chain reaction (qRT-PCR) analysis. All specific primers were designed using the Primer-Blast tool^2^. Reverse transcription and qRT-PCR were conducted following standard procedures. Three technical replicates and three biological replicates were performed, and the _ΔΔ_Ct algorithm was used for calculating relative gene expression (Pfaffl, 2001). The endogenous reference gene was *GhY8991* in TM-1 and *GbY8991* in Hai7124. Sequences of the primer pairs used in this study are listed in Supplementary Table 2.

## Results

### Identification of Genes With Subgenome-Biased Expression in *Gossypium* Species

In terms of the three fiber quality traits elongation rate, fiber length, and fiber strength, the *G. barbadense* cultivars were significantly superior to the *G. hirsutum* cultivars (*P* < 3.20 × 10^–2^, *T*-test); in contrast, in terms of the yield trait single boll weight, the *G. hirsutum* cultivars were significantly better than the *G. barbadense* cultivars (*P* < 1.10 × 10^–2^, *T*-test) (Supplementary Figure 1). To reveal the underlying cause of this distinct interspecies difference in fiber quality, we sequenced samples collected from four *G. hirsutum* and three *G. barbadense* cultivars at specific fiber developmental stages, namely 10 and 20 DPA. A total of 36 libraries were constructed for *G. hirsutum* samples, with an average of 49.41 million raw reads per library and a mapping rate of 94.40%; meanwhile, a total of 32 libraries were constructed for *G. barbadense* samples, with an average of 49.26 million raw reads per library (Supplementary Table 1). Similarity analysis using the Pearson correlation coefficient was conducted to compare every biological replicate, and returned correlation values of more than 84%, indicating good repeatability (Supplementary Figure 2). PCA further confirmed the commonality of gene expression patterns between different stages in *G. hirsutum* and *G. barbadense* (Supplementary Figure 3).

We further analyzed global expression bias between the A and D subgenomes by comparing the average FPKM values of homologous pairs in *G. hirsutum* and *G. barbadense*. This revealed an obvious tendency in that expression of 15.90–37.96% of genes was biased toward either the A or D subgenome, with more being biased toward the D subgenome (Table 1). Specifically, in J220 at 10 DPA, 1733 genes were biased toward the A subgenome and 1924 genes to the D subgenome (*P* = 3.90 × 10^–4^, Fisher’s exact test), accounting for 25.06% of total expressed genes. Similarly, in 3–79 at 20 DPA, 28.45% of genes were biased toward either the A or D subgenome, with most being biased toward the D subgenome (*P* = 1.08 × 10^–2^, Fisher’s exact test). In contrast, XLZ42 at 10 DPA showed expression bias toward the A subgenome for 3493 genes, significantly more than exhibited bias toward the D subgenome. These distinct expression biases may result from the differing, complex background of these cultivars’ respective breeding histories (Huang, 2007).

**TABLE 1:** Homologous expression bias of gene pairs.

Material	Total expressed gene pairs	Total biased-expressed gene pairs	A_*t*_ > D_*t*_	A_*t*_ < D_*t*_	Bias/Total (%)	*p*-value	Signifi-cance^1^	Bias toward
***G. hirsutum* 10 DPA**								
J220	14595	3657	1733	1924	25.06%	3.898E-04	**	Dt
TM-1	15393	2448	1184	1264	15.90%	4.803E-02	*	Dt
4005	15270	3356	1558	1798	21.98%	6.090E-06	**	Dt
XLZ42	13842	5254	3493	1761	37.96%	1.100E-16	**	At
***G. hirsutum* 20 DPA**								
J220	12796	3425	1641	1784	26.77%	4.562E-03	**	Dt
TM-1	13615	2768	1305	1463	20.33%	8.190E-04	**	Dt
4005	13560	3228	1535	1693	23.81%	1.617E-03	**	Dt
XLZ42	12284	3363	1595	1768	27.38%	7.040E-04	**	Dt
***G. barbadense* 10 DPA**								
Hai7124	15118	3319	1497	1822	21.95%	1.226E-09	**	Dt
R4-4	14902	3646	1677	1969	24.47%	1.327E-07	**	Dt
3-79	13993	2720	1242	1478	19.44%	1.044E-06	**	Dt
***G. barbadense* 20 DPA**								
Hai7124	14203	2362	1140	1222	16.63%	4.087E-02	*	Dt
R4-4	12861	3353	1575	1778	26.07%	9.140E-05	**	Dt
3-79	12536	3566	1719	1847	28.45%	1.083E-02	*	Dt

^1^ * indicates p < 0.05; ** indicates p < 0.01, Fisher’s exact test.

*At: A subgenome; Dt: D subgenome.*

### Inter- and Intra-Specific Comparison of Subgenome-Biased Genes

**FIGURE 1:**
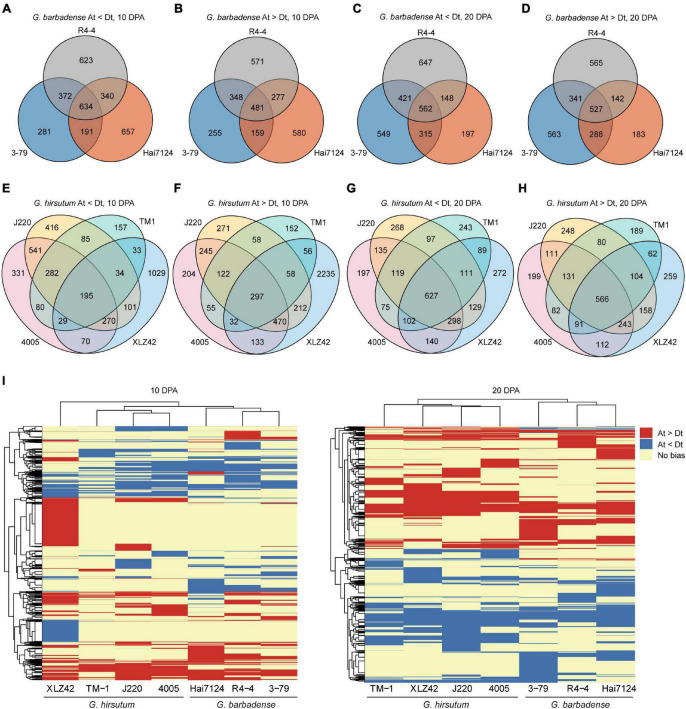
Differences in homologous expression bias among tested cultivars. **(A–H)** Venn diagrams of gene pairs with homologous expression bias. Each circle represents one cultivar, number of genes with biased expression, and detailed information is denoted below each graph. **(I)** Heatmap of biased genes at 10 and 20 DPA. Red indicates bias toward the A subgenome, blue bias toward the D subgenome, and yellow no expression bias (Gene pairs showing no bias in all cultivars are omitted).

To discover inter- and intra-specific subgenome expression biases, we compared the various cultivars and determined whether biased genes were shared or specific in 10- and 20-DPA fiber tissues. In all three *G. barbadense* cultivars at 10 DPA, 634 genes showed expression bias toward the D subgenome, while 481 genes were biased toward the A subgenome (Figures 1A,B). At 20 DPA, the corresponding counts were 527 and 562 genes respectively biased toward the A and D subgenomes (Figures 1C,D). In other words, genes biased toward the D subgenome were more shared at 10 DPA than that at 20 DPA, and the opposite was true for A-biased genes. Meanwhile, across the four cultivars of *G. hirsutum*, 195 and 297 genes, respectively showed common bias at 10 DPA toward the D and A subgenomes (Figures 1E,F), while the corresponding counts at 20 DPA were 627 and 566 genes (Figures 1G,H). These results indicate the presence of common and specific subgenome-biased genes in both *G. hirsutum* and *G. barbadense*.

To compare inter-specific patterns of bias between *G. hirsutum* and *G. barbadense*, we constructed a heatmap of biased gene pairs at 10 and 20 DPA. Hierarchical clustering revealed huge differences in expression bias pattern between *G. hirsutum* and *G. barbadense*, including genes having consistent bias, distinct bias to different subgenomes, or no bias. For instance, considerable numbers of A-biased genes were detected in *G. hirsutum* (XLZ42) at 10 DPA and many D-biased genes in *G. barbadense* (3–79) at 20 DPA (Figure 1I).

In total, 461 and 838 gene pairs with extreme expression bias patterns were detected at 10 and 20 DPA, respectively (Table 2 and Supplementary Table 3). For instance, 78 genes at 10 DPA and 106 genes at 20 DPA showed strict bias toward the A subgenome in *G. hirsutum*, but no biased pattern in *G. barbadense*. Several genes exhibited opposite patterns between species, including two genes at 10 DPA and three genes at 20 DPA biased to the A subgenome in *G. hirsutum* and the D subgenome in *G. barbadense*. For example, *GhPPR* (pentatricopeptide repeat, *GH_A05G0653*) was predominantly expressed from the A subgenome at 20 DPA in all four *G. hirsutum* cultivars but showed no bias in the three *G. barbadense* cultivars. Another gene, *GhHAD* (haloacid dehalogenase-like hydrolase, *GH_A05G0047*), was expressed more from the D subgenome at 20 DPA in all four *G. hirsutum* cultivars, but from the A subgenome in the three *G. barbadense* cultivars. Meanwhile, the gene *GhMAPK1* (mitogen-activated protein kinase 1, *GH_A05G1909*) was expressed from the D subgenome at 20 DPA in the three *G. barbadense* cultivars but showed no bias in the four *G. hirsutum* cultivars. Overall, our results suggest that the biased expression patterns of homologous gene pairs were changed after the independent domestications of *G. hirsutum* and *G. barbadense*, which may contribute to their different fiber qualities.

**TABLE 2:** Summary of homologous gene pairs with extreme expression bias patterns.

Biased in *G. hirsutum*	Biased in *G. barbadense*	Counts at 10 DPA	Counts at 20 DPA
A_*t*_ > D_*t*_	No bias	78	106
A_*t*_ > D_*t*_	A_*t*_ < D_*t*_	2	3
A_*t*_ > D_*t*_	A_*t*_ > D_*t*_	72	163
A_*t*_ < D_*t*_	No bias	51	132
A_*t*_ < D_*t*_	A_*t*_ > D_*t*_	0	4
A_*t*_ < D_*t*_	A_*t*_ < D_*t*_	59	189
No bias	A_*t*_ > D_*t*_	77	115
No bias	A_*t*_ < D_*t*_	122	126

*At: A subgenome; Dt: D subgenome.*

*This table shows extreme cases. For example, A subgenome > D subgenome means the gene is expressed from the A subgenome in all three G. barbadense or four G. hirsutum cultivars.*

### Temporally Postponed Expression of Fiber-Related Genes in *Gossypium* Species

**FIGURE 2:**
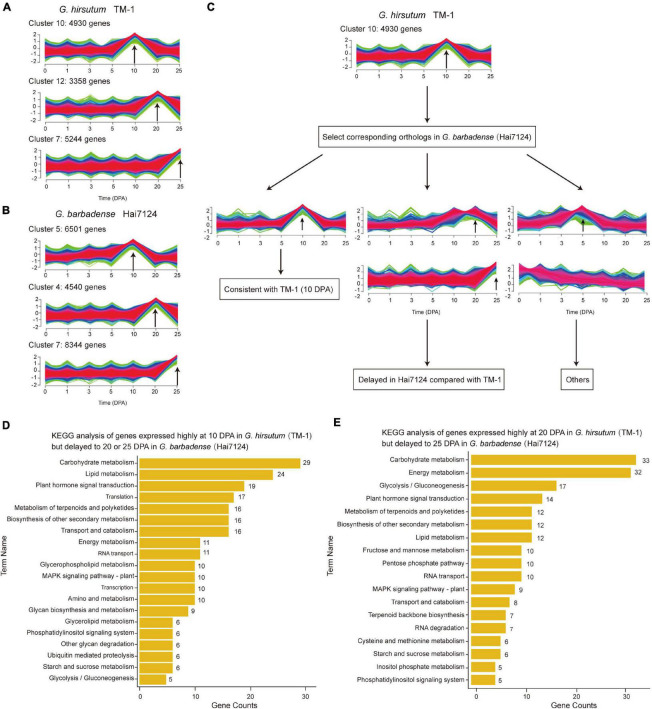
Comparison of temporal expression patterns in TM-1 and Hai7124. **(A,B)** Clusters associated with essential fiber development stages (10, 20, and 25 DPA) in TM-1 and Hai7124, respectively. Each bar indicates one cluster. *X*-axis represents time (0–25 DPA) and *Y*-axis the magnitude of expression change. Numbers denote the count of genes in each cluster. **(C)** Schematic diagram of differences in temporal expression, taking 10 DPA as an example. **(D,E)** KEGG analysis of genes with delayed expression patterns. *X*-axis represents the number of genes in each term and *Y*-axis the term names. Numbers alongside denote the count of genes in each term.

The extended elongation phase in *G. barbadense* Hai7124 (5–30 DPA) most likely leads to development of longer fibers as compared to *G. hirsutum* TM-1 (Hu et al., 2019), as is characteristic of *G. barbadense* vs. *G. hirsutum*. Here, we systematically identified fiber related genes whose expression was temporally postponed. During the fiber initiation stage from 0 to 5 DPA, the total number of expressed genes in Hai7124 was significantly more than that in TM-1, while during the elongation and second cell wall biosynthesis stage from 10 to 25 DPA, the total number of expressed genes in TM-1 began to increase, ultimately surpassing the number expressed in Hai7124 (Supplementary Figure 4). By comparing temporal differences in expression between TM-1 and Hai7124 at 10, 20, and 25 DPA, we identified 4930, 3358, and 5244 genes expressed predominantly in TM-1, and 6501, 4540, and 8344 genes expressed predominantly in Hai7124 (Supplementary Figures 5A,B and Figures 2A,B). The genes predominantly expressed at 10 DPA were mainly enriched in translation factor activity, translation regulator activity, and response to external stimulus; meanwhile, those predominantly expressed at 20 DPA were mainly enriched in catalytic activity, lipid metabolic process, carbohydrate metabolic process, and signal transduction. These enrichments indicated these genes as representing dynamic adjustment to influence various life activities of fiber cells and systematically promote fiber formation. With respect to enriched GO terms, *G. hirsutum* and *G. barbadense* were almost equivalent at 20 DPA. In contrast, for genes predominantly expressed at 10 DPA, TM-1 evidenced distinct enrichment in classes relating to response to stress, chemical stimulus, and endogenous stimulus, while Hai7124 exhibited specific enrichment of the terms about transcription activity and secondary metabolic process (Supplementary Figures 5C,D).

These predominantly expressed genes also revealed several patterns of temporally postponed gene expression between Hai7124 and TM-1 (Figure 2C). A total of 2128 genes exhibited consistent expression patterns between TM-1 and Hai7124 at any timepoint (643 at 10 DPA, 621 at 20 DPA, and 864 at 25 DPA). Conversely, 6791 genes revealed distinct expression patterns, including 625 genes predominantly expressed during the fiber elongation period of TM-1 but delayed to the secondary wall thickening period in Hai7124. For instance, 346 genes were predominantly expressed at 10 DPA in TM-1 but postponed to 20 DPA in Hai7124, while 279 genes were predominantly expressed at 10 DPA in TM-1 but postponed to 25 DPA in Hai7124 (Supplementary Table 4). Annotations from KEGG revealed these genes as mainly participating in carbohydrate metabolism, lipid metabolism, plant hormone signal transduction, starch and sucrose metabolism, and other such pathways (Figure 2D). Similarly, a total of 367 genes were identified as predominantly expressed at 20 DPA in TM-1 but postponed to 25 DPA in Hai7124 (Supplementary Table 5), and these genes participated in carbohydrate metabolism, glycolysis/gluconeogenesis, plant hormone signal transduction, and lipid metabolism (Figure 2E). These results are consistent with previous reports that various aspects of metabolism are involved in fiber development, particularly through synthesis of the primary and second cell walls, and that multiple plant hormones play pivotal roles in fiber cell development (Xiao et al., 2019; Huang et al., 2021).

### Temporal Postponement of Metabolism Pathways in Fiber Development

**FIGURE 3:**
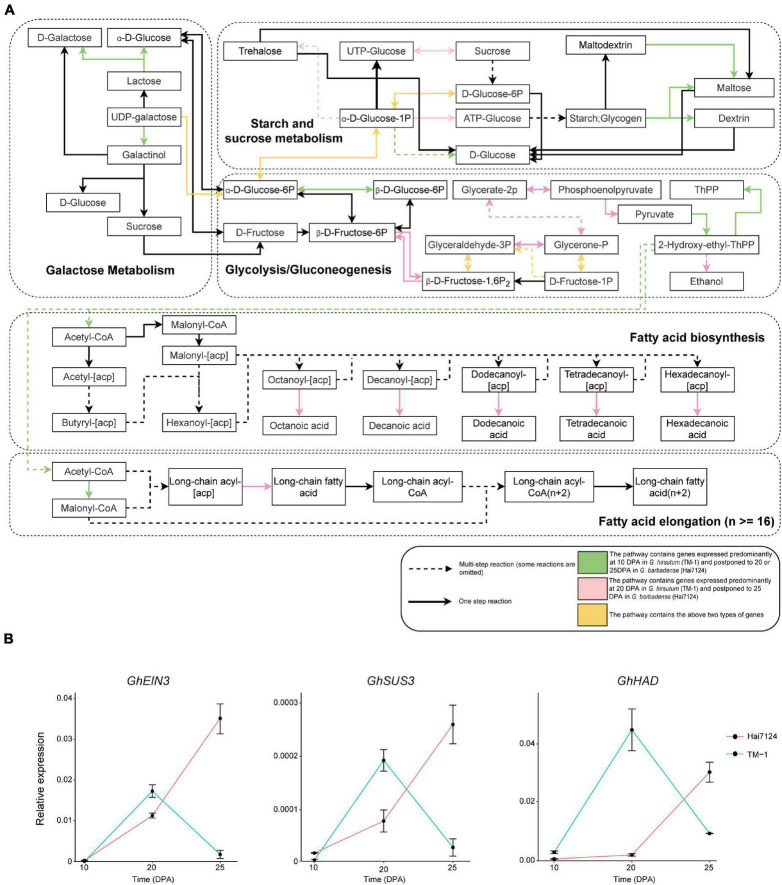
Biased expression in lipid and carbohydrate metabolism pathways and validation of selected genes. **(A)** Diagram of lipid and carbohydrate metabolism pathways. Rectangles represent the substrate or product of a biochemical reaction. Arrows represent biochemical reactions. Green indicates a pathway containing genes expressed predominantly at 10 DPA in TM-1 and postponed to 20 DPA in Hai7124. Pink indicates a pathway containing genes expressed predominantly at 20 DPA in TM-1 and postponed to 25 DPA in Hai7124. Yellow indicates a pathway containing both types of genes. Some reactions are omitted. **(B)** qRT-PCR validation of *GhEIN3*, *GhSUS3*, and *GhHAD* in fibers from TM-1 and Hai7124 plants at 10, 20, and 25 DPA. Each value represents the mean ± s.e.m.

A number of vital genes were obviously postponed in Hai7124 relative to TM-1, including genes that participate in key steps of galactose metabolism, glycolysis/gluconeogenesis, and fatty acid biosynthesis (Figure 3A). In addition, similar results were found in plant hormone signal transduction pathways involving auxin, cytokine, gibberellin, ethylene, brassinosteroids, and jasmonic acid (Supplementary Figure 6). For instance, two *EXPA8* genes (member of Alpha-Expansin gene family, *GB_D13G0874* and *GB_A13G0955*) were expressed predominantly at 10 DPA in TM-1 and postponed to 20 DPA in Hai7124; the products of these genes convert malate to oxaloacetic acid and generate pyruvate and acetyl-CoA, substrates for fatty acid synthesis. A gene involved in fructose metabolism, *GB_A10G0497* encoding a glucose-6-phosphate 1-epimerase, was expressed predominantly at 10 DPA in TM-1 and postponed to 25 DPA in Hai7124. Moreover, two other genes involved in fructose and mannose metabolism, *GB_A05G3796* and *GB_A05G3098* encoding a fructose-bisphosphate aldolase, were expressed predominantly at 10 DPA in TM-1 and postponed to 25 DPA in Hai7124.

Concerning hormone-related genes, *GB_A05G0554* and *GB_D08G2840* encoding auxin-responsive proteins were expressed predominantly at 20 DPA in TM-1 and postponed to 25 DPA in Hai7124. Another two genes, *GB_A02G0427* and *GB_D02G0455* encoding ethylene-insensitive proteins, were expressed predominantly at 20 DPA in TM-1 and postponed to 25 DPA in Hai7124; the protein products of these genes were reported to promote root hair growth in Arabidopsis (Feng et al., 2017). In addition, the postponed genes encompassed a total of 123 transcription factors (TFs), including 13 ERFs, 9 NACs, 9 WRKYs, 6 bHLHs, 6 DOFs, 5 MYBs, and 2 WOXs. Three ortholog pairs were validated by qRT-PCR: *GhSUS3_*D_*T*_ [*GH_D08G1434*] and *GbSUS3_*D_*T*_ [*GB_D08G1489*], *GhHAD_*A_*T*_ [*GH_A06G0396*] and *GbHAD_*A_*T*_ [*GB_A06G0402*], *GhEIN3_*A_*T*_ [*GH_A02G0431*] and *GbEIN3_*A_*T*_ [*GB_A02G0427*]; this assay confirmed these genes as having postponed expression in Hai7124 compared to TM-1 (Figure 3B). Hence, we propose that this pattern of temporal postponement of expression during fiber development in *G. barbadense* relative to *G. hirsutum* is an essential factor in the development of extra-long fibers.

### Interspecific Asymmetry of Fiber Development Co-expression Networks and Hub Genes

**FIGURE 4:**
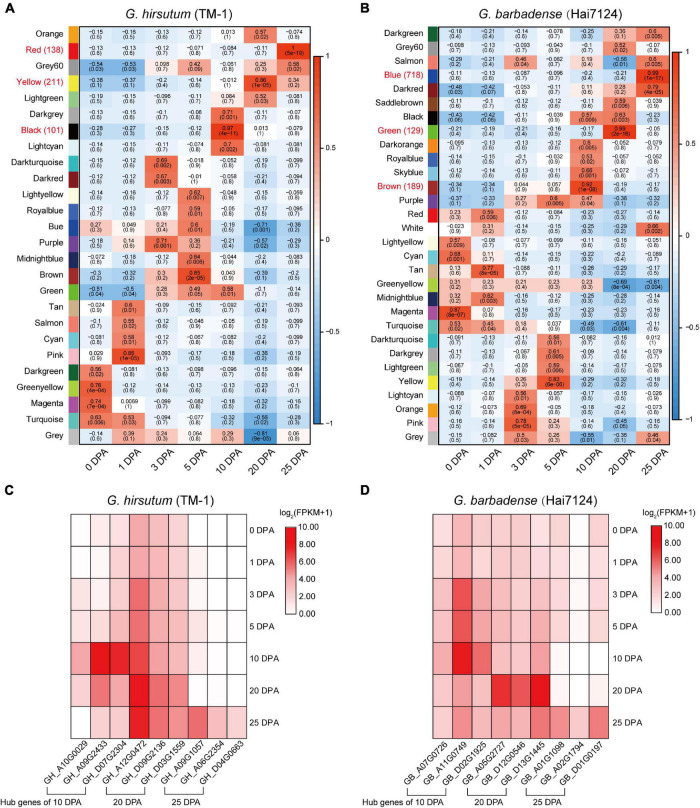
Weighted gene co-expression network analysis in Hai7124 and TM-1. **(A,B)** Module-trait relationships of Hai7124 and TM-1, respectively. Each row represents a module, and each column one fiber developmental stage; in each square are denoted the corresponding correlation coefficient and *p*-value. Red names indicate the modules most associated with timepoints 10, 20, or 25 DPA, and the following number tallies TFs in the module. **(C)** Expression of hub genes in the modules most closely related to 10, 20, or 25 DPA in TM-1. **(D)** Expression of hub genes in the modules most closely related to 10, 20, or 25 DPA in Hai7124. The base-2 logarithm of FPKM value plus 1 is utilized. Stage associations of hub genes are denoted below.

In order to further understand the relationship between gene regulation and fiber development, and to identify genes specifically related to fiber quality, WGCNA was used to separately generate co-expression networks of gene orthologs in TM-1 and Hai7124 (Figure 4). Hierarchical clustering was used to construct the topology overlap matrix, and dynamic cut modules that characterized similar expression patterns were merged. In total, 26 and 30 modules were, respectively identified based on gene expression profiles in TM-1 and Hai7124. In TM-1, each critical period of fiber development had a corresponding module, and the genes in different modules related to the different stages of fiber development (Figure 4A). The black module, which corresponded to 10 DPA, was comprised of 1344 genes including 100 TFs. The yellow module (3700 genes, including 211 TFs) was dramatically associated with 20 DPA, the period of transitional cell-wall remodeling and biosynthesis of the winding layer. Finally, the red module (1430 genes, including 137 TFs) was highly related to the 25 DPA period, the key stage of secondary wall formation (Supplementary Table 6).

Meanwhile, Hai7124 exhibited distinct modules for each timepoint, but also an overlapping module. The brown module was specific to 10 DPA and consisted of 4948 genes, including 189 TFs. The green module (2400 genes, including 129 TFs) was highly associated with 20 DPA, the period of transitional cell-wall remodeling. The blue module (5046 genes, including 718 TFs) was highly associated with 25 DPA, the key period of secondary wall shaping. However, genes in the black module were expressed at both 10 and 20 DPA; the products of these genes were involved in stages of primary cell wall (PCW) development and also in the transition to SCW growth (Figure 4B and Supplementary Table 7).

Regarding TFs specifically, each timepoint exhibited distinct profiles for *G. hirsutum* and *G. barbadense* in terms of the number and category of TFs expressed. For example, at 10 DPA, 189 TFs were expressed in Hai7124 vs. 100 TFs in TM-1. Three of those expressed in TM-1 were specific, namely E2F/DP, VOZ, and WOX; meanwhile, 12 TFs were specific to Hai7124 at 10 DPA, including AP2, ARF, DBB, FAR1, G2-like, HB-other, M-type, NF-YC, Nin-like, RAV, SBP, and ZF-HD (Supplementary Tables 6, 7). At 25 DPA, 718 TFs were expressed in Hai7124, significantly more than the 137 TFs detected in TM-1 at the same period. These results reveal a prolonged contribution of the TFs in *G. barbadense* to fiber development, consistent with a previous finding that postponed expression of *GbVIN1*, *GbNHX1*, and *GbTST1* in *G. barbadense* leads to the development of longer fibers (Hu et al., 2019).

In WGCNA, hub genes are identified by sorting K_*ME*_ values. Given that 10, 20, and 25 DPA represent critical stages in fiber development, we selected as hub genes for each module the three genes having the highest K_*ME*_ values (Figure 4C and Supplementary Table 8). In TM-1, hub genes in the black module (10 DPA) encoded indole-3-acetic acid inducible protein, indoleacetic acid-induced protein, and C6-N-acetylglucosaminyltransferase family protein. Meanwhile, hub genes associated with the yellow module (20 DPA) annotated as N-acetylglucosamine-1-phosphate uridylyltransferase, membrane protein, and p-loop containing nucleoside triphosphate hydrolases superfamily protein. In the red module (25 DPA), hub genes encoded serine carboxypeptidase-like acyltransferase, DUF506 family protein, and glycerol-3-phosphate acyltransferase. Notably, glycerol-3-phosphate acyltransferase is known to be a rate-limiting enzyme in the triacylglycerol biosynthesis of glycerolipids, which is extensively involved in the elongation of cotton fibers.

For Hai7124, hub genes in the brown module (10 DPA) mainly encoded early nodulin-like, alpha/beta-hydrolase superfamily, and cytochrome P450-related proteins (Figure 4D). Those in the green module (20 DPA) annotated as belonging to the S-adenosyl-L-methionine-dependent methyltransferase superfamily, major facilitator superfamily, and alpha/beta-hydrolase superfamily. The blue module associated with 25 DPA featured hub genes encoding filament-like protein, GBF’s pro-rich region-interacting factor, and a GMA12/MNN10 family protein.

**FIGURE 5:**
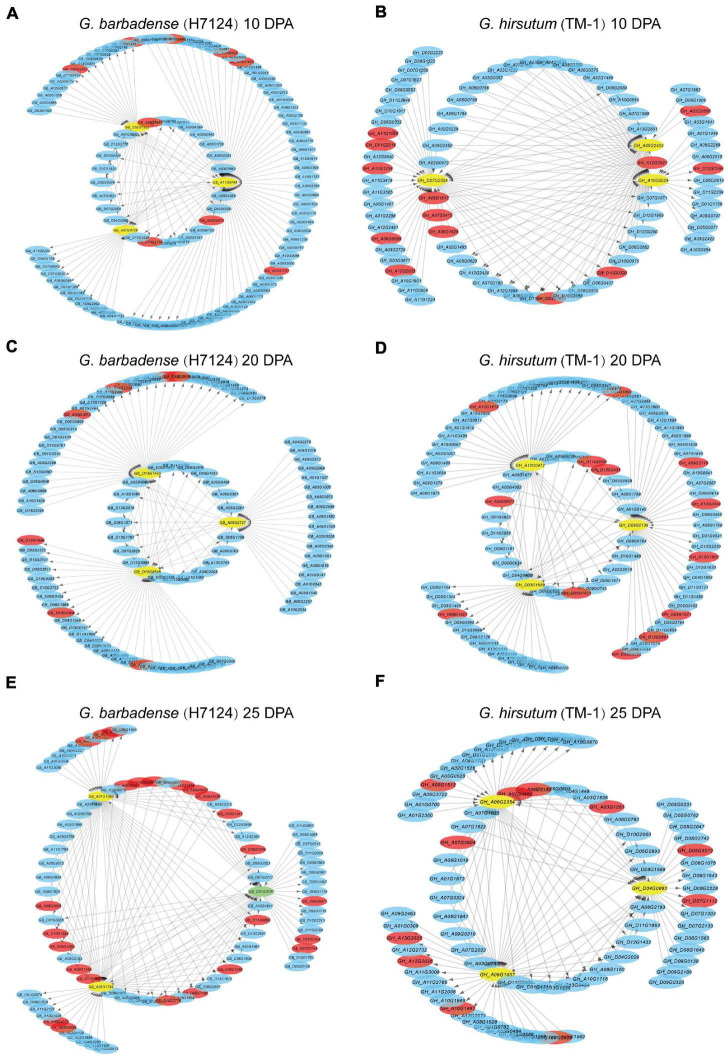
Interaction networks of Hai7124 **(A,C,E)** and TM-1 **(B,D,F)** genes expressed at 10, 20, and 25 DPA, respectively. Hub genes are indicated by yellow circles and TFs by red bars. Green circles indicate those that are both hub gene and transcription factor. Arrows indicate the action of one gene on another.

The correlation networks of the high-weight value pairs in each module were visualized using gene interaction edges with high weights (Figure 5). We further compared expression timing of hub genes from the Hai7124 brown module (10 DPA), green module (20 DPA), and blue module (25 DPA) with their corresponding orthologs in TM-1 (Supplementary Table 9). Orthologs of three hub genes in the Hai7124 brown module (10 DPA) were associated with the TM-1 green module (5 and 10 DPA). Meanwhile, hub genes in the Hai7124 green module (20 DPA) corresponded to orthologs in the TM-1 yellow module (also highly specific to 20 DPA). However, three hub genes in the Hai7124 blue module (25 DPA; *GB_A01G1098*, *GB_D01G0197*, and *GB_A02G1794*) corresponded to genes associated with 1 and 5 DPA timepoints in TM-1.

Similar steps were implemented to compare hub genes of TM-1 at 10, 20, and 25 DPA to their corresponding orthologs in Hai7124. For instance, *GH_A10G0029*, as one of three TM-1 hub genes at 10 DPA, was associated with the 25 DPA timepoint in Hai7124 (Supplementary Table 9). Overall, the asymmetry of co-expression networks and hub genes in *G. barbadense* and *G. hirsutum* illuminate their roles in the distinct fiber quality developed by these species after allotetraploid cotton formation.

## Discussion

### Homologous Genes Show Both Intra- and Inter-Species Expression Bias

Polyploidy is a fundamental process in plant evolution that may have genome-wide impacts on gene expression variation. In this study, homologous expression bias was investigated in samples from three *G. barbadense* and four *G. hirsutum* lines. A previous study reported no evidence of genome-wide expression bias between homologous gene pairs in in different accessions, while in *G. hirsutum* TM-1, 20–40% of homologous gene pairs showed bias toward the A- or D-homolog (Zhang et al., 2015). In our study, 15.90–37.96% of gene pairs showed expression bias toward the A or D subgenome, with bias toward the D subgenome predominating; this finding will benefit our understanding of the mechanisms of fiber development in *G. hirsutum* and *G. barbadense*. We also observed some homologs to exhibit completely opposite patterns of expression bias between the two species. Interestingly, this included three genes encoding hydrolase superfamily proteins (*GH_A05G0047*, *GH_A07G1580*, and *GH_A01G0676*), whose expression at 20 DPA was biased toward the D subgenome in *G. hirsutum* and toward the A subgenome in *G. barbadense*. Similarly, a gene encoding NAD(P)-linked oxidoreductase superfamily protein (*GH_A13G1238*) was at both 10 and 20 DPA more expressed from the A subgenome in *G. hirsutum* and from the D subgenome in *G. barbadense*. Deeper investigation should be conducted to elucidate the potential divergent functions these genes might carry out.

### Crucial Genes and Transcription Factors Involved in Cotton Fiber Development

At present, breeding of new cultivars that incorporate the advantages of both allotetraploid cottons has become a top priority. Advances in transgenic technology have enabled elucidation of functionality for an increasing number of genes involved in fiber development. For example, the TF *GhTCP4* has been reported to function antagonistically with a homeobox-containing factor, *GhHOX3*, to regulate cell elongation, leading to the establishment of temporal control over the transition of fiber cells to the secondary wall stage (Cao et al., 2020). A cotton JAZ protein, *GhJAZ2*, was identified to regulate the JA signaling pathway by interacting with *GhMYB25-like*, *GhGL1*, *GhMYC2*, *GhWD40*, and *GhJI1*, thereby acting as a major transcriptional repressor during the initiation of cotton lint and fuzz fibers (Hu et al., 2016). Some chemical factors have been reported to have decisive roles in regulating fiber elongation and development, such as calcium (Huang et al., 2008), actin (Preuss et al., 2004; Huang et al., 2013; Zhang et al., 2017), BRs (Sun et al., 2005; Lu et al., 2018), and ethylene (Shi et al., 2006; Qin et al., 2007). Notably, the elongation period is an important developmental stage that determines mature cotton fiber length, with the fiber at this stage reaching about 80% of its mature size. In the present study, genes expressed in Hai7124 at 20 DPA were enriched for the term “Ubiquitin mediated proteolysis,” and those expressed at 25 DPA were enriched for “Terpenoid backbone biosynthesis,” “Metabolism of terpenoids and polyketides,” and “Diterpenoid biosynthesis.” In TM-1, genes expressed at 20 DPA were enriched for “Inositol phosphate metabolism,” and those at 25 DPA for “Metabolism of terpenoids and polyketides.” Chemical factors such as ubiquitin, terpenoids, and inositol phosphate may make essential contributions to fiber development (Champagne and Boutry, 2016; Feng et al., 2018; Ma et al., 2019). Moreover, the postponed expression pattern was exhibited by many genes involved in fatty acid biosynthesis, carbohydrate metabolism, and hormone signal transduction; this altered expression might be responsible for the differences of fiber quality between TM-1 and Hai7124.

### Asymmetric Hub Genes Possibly Contribute to Divergent Fiber Quality

Weighted gene co-expression network analysis was applied to identify significant modules consisting of genes associated with specific fiber developmental stages and to select hub genes, which occupy vital roles in co-expression networks. We focused on the modules respectively associated with 10, 20, and 25 DPA timepoints to better understand co-expression network interactions during rapid fiber elongation and secondary wall thickening. Numerous TFs were identified in each module, which might reflect differences in transcription activity between TM-1 and Hai7124 (Supplementary Tables 6, 7). For example, in TM-1, TFs identified at 25 DPA included eleven *bHLHs* that may promote fiber elongation by modulating brassinosteroid biosynthesis and signaling (Liu et al., 2020), 11 *ERFs* involved in response to salt/drought and ABA (Ma et al., 2017), 12 MYBs, and 17 NACs. Meanwhile, TFs expressed in Hai7124 included a total of 64 bHLHs, 51 ERFs, 59 MYBs, and 49 NACs, indicating the possibility that more transcription activity occurs in Hai7124 fibers at 25 DPA. Moreover, the asymmetric co-expression of network and hub genes in TM-1 and Hai7124 might indicate a key aspect of differential fiber development. All told, we not only identified consistent patterns of expression and regulation networks between *G. hirsutum* and *G. barbadense*, but also found evidence supporting a pattern of temporally postponed expression closely related to the species’ different fiber qualities. Our study also provides a large number of candidate genes grouped in different expression modules as a substantial resource for cotton fiber improvement.

## Conclusion

Based on RNA-seq of fiber samples from four *G. hirsutum* and three *G. barbadense* cultivars, we found that 15.90–37.96% of differentially expressed genes showed biased expression toward the A or D subgenome from each species. We further found 6791 genes demonstrated temporal differences in expression. Among them, 346 genes predominantly expressed at 10 DPA in *G. hirsutum* (TM-1) but postponed to 20 DPA in *G. barbadense* (Hai7124), and 367 genes predominantly expressed at 20 DPA in TM-1 but postponed to 25 DPA in Hai7124. These postponed genes enriched in carbohydrate metabolism, lipid metabolism, plant hormone signal, starch and sucrose metabolism. Co-expressed analysis showed that the hub genes associated in fiber development showed asymmetric expression between TM-1 and Hai7124.

## Data Availability Statement

The original contributions presented in the study are publicly available. This data can be found here: The TM-1 (v2.1) and Hai7124 (v1.1) assembly genomes and annotation data are available at http://cotton.zju.edu.cn. Illumina RNA-Seq data are available at the Sequence Read Archive under accession numbers PRJNA490626 (TM-1 and Hai7124) and PRJNA697906 (J220, 4005, XLZ42, and R4-4, 3-79).

## Author Contributions

LF conceptualized the research program. HM, BQ, and ZH finished the analysis of this study and wrote the manuscript. TZ hao, MG, JH, XG, and YH designed the qRT-PCR experiment and finished the operation. JZ planted the material and collected the fiber. LF, TZhang, HM, and BQ revised the manuscript. All authors discussed the results, commented on the manuscript and approved the submitted version.

## Conflict of Interest

The authors declare that the research was conducted in the absence of any commercial or financial relationships that could be construed as a potential conflict of interest.

## Publisher’s Note

All claims expressed in this article are solely those of the authors and do not necessarily represent those of their affiliated organizations, or those of the publisher, the editors and the reviewers. Any product that may be evaluated in this article, or claim that may be made by its manufacturer, is not guaranteed or endorsed by the publisher.

## Footnotes

1
https://www.kegg.jp/kegg/mapper.html

2
http://www.ncbi.nlm.nih.gov/tools/primer-blast
