# Supplementary material for: Subgenome Bias and Temporal Postponement of Gene Expression Contributes to the Distinctions of Fiber Quality in Gossypium Species
Source: Front Plant Sci. 2021 Dec 23;12:819679. doi: 10.3389/fpls.2021.819679 (PMC8733733; doi:10.3389/fpls.2021.819679)
Supplement: Supplementary file 1 [file Data_Sheet_1.PDF]

Supplementary Materials:

Figure S1. Comparison of yield and fiber quality traits between *G. hirsutum* and *G. barbadense*.

Figure S2. Repeatability verification of all samples from *G. barbadense* and *G. hirsutum*.

Figure S3. Principal component analysis of samples used in this study.

Figure S4. Numbers of differentially expressed genes in *G. hirsutum* (TM-1) and *G. barbadense* (Hai7124).

Figure S5. Clustering results for *G. barbadense* (Hai7124) and *G. hirsutum* (TM-1) with GO analysis.

Figure S6. Participation of postponed genes in plant hormone signaling pathways.

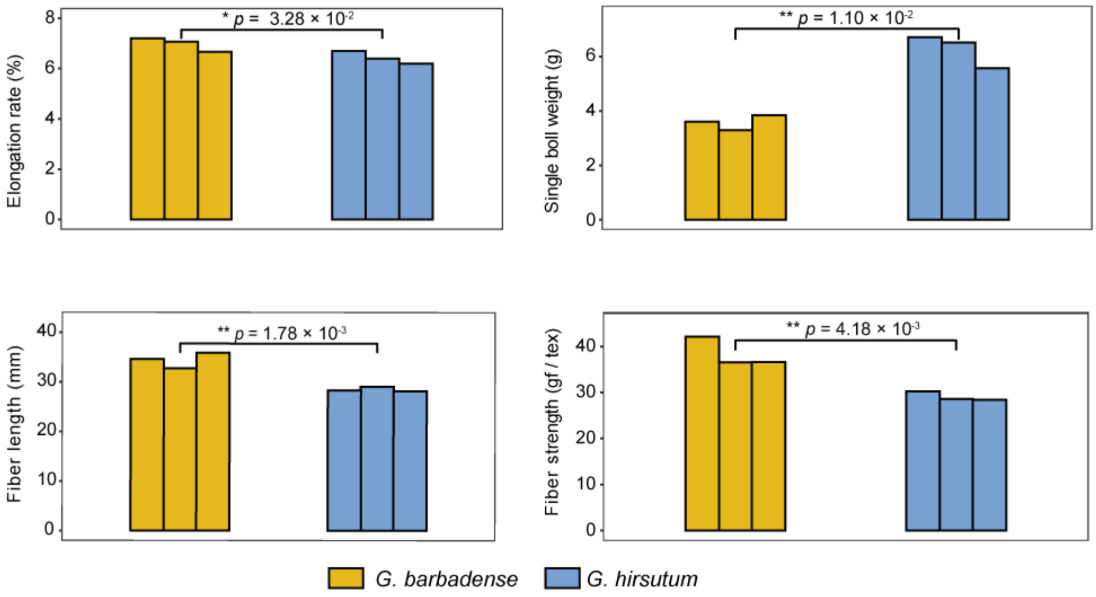

Figure S1. Yield and fiber quality traits comparison between *G. hirsutum* and *G. barbadense*. The bar plot was conducted using average phenotype value. **: $p < 0.01$; *: $p < 0.05$; NS: no significance, t-test (J220 lacked phenotype data).

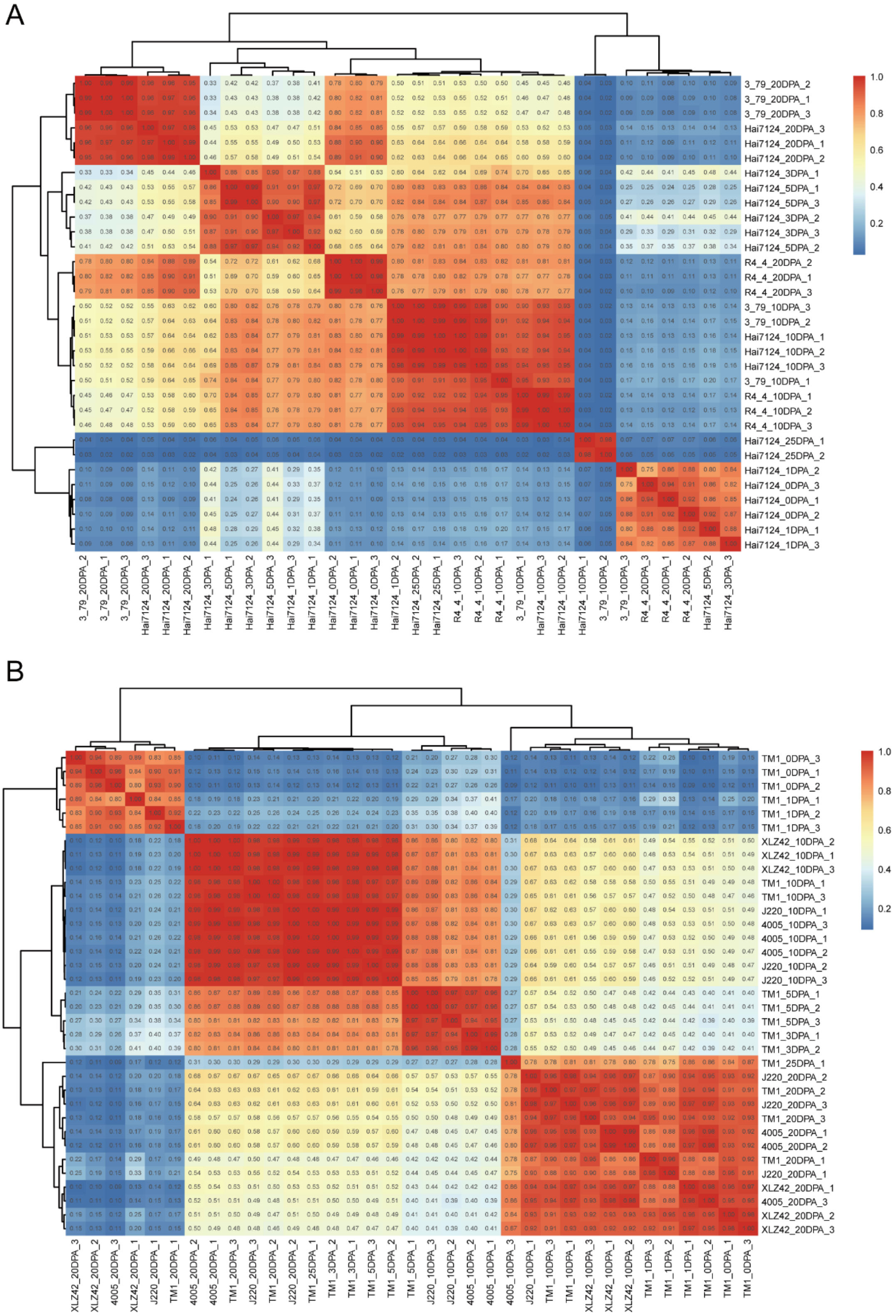

Figure S2. Repeatability verification of all samples from *G. barbadense* and *G. hirsutum*. (A) Repeatability verification of samples from *G. barbadense* (3-79, R4_4, Hai7124). **(B)** Repeatability verification of samples from *G. hirsutum* (TM-1, J220, 4005, XLZ42).

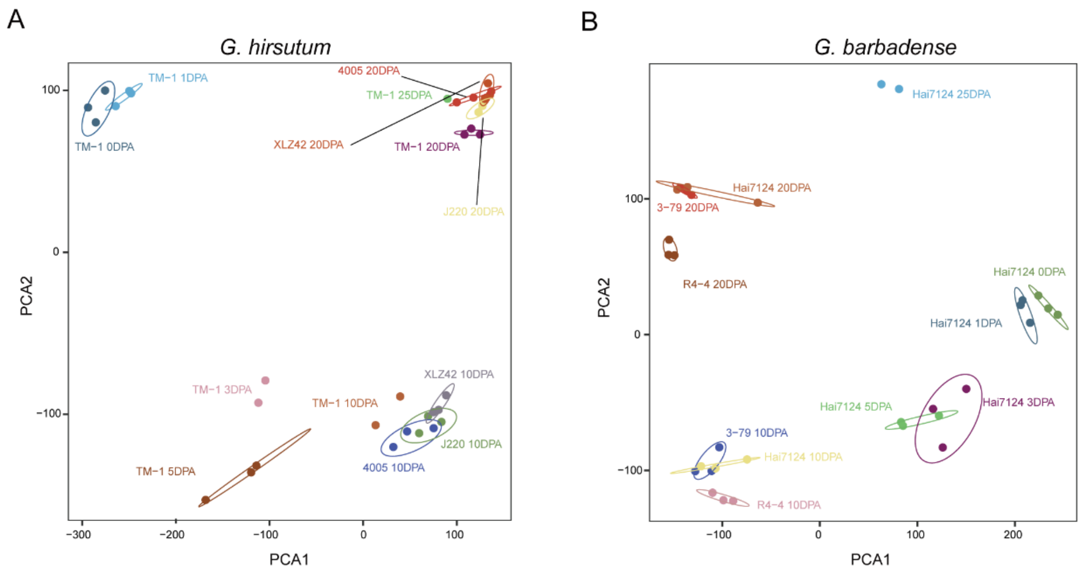

Figure S3. Principal component analysis for sequenced samples of *G. hirsutum* (A) and *G. barbadense* (B) used in this study.

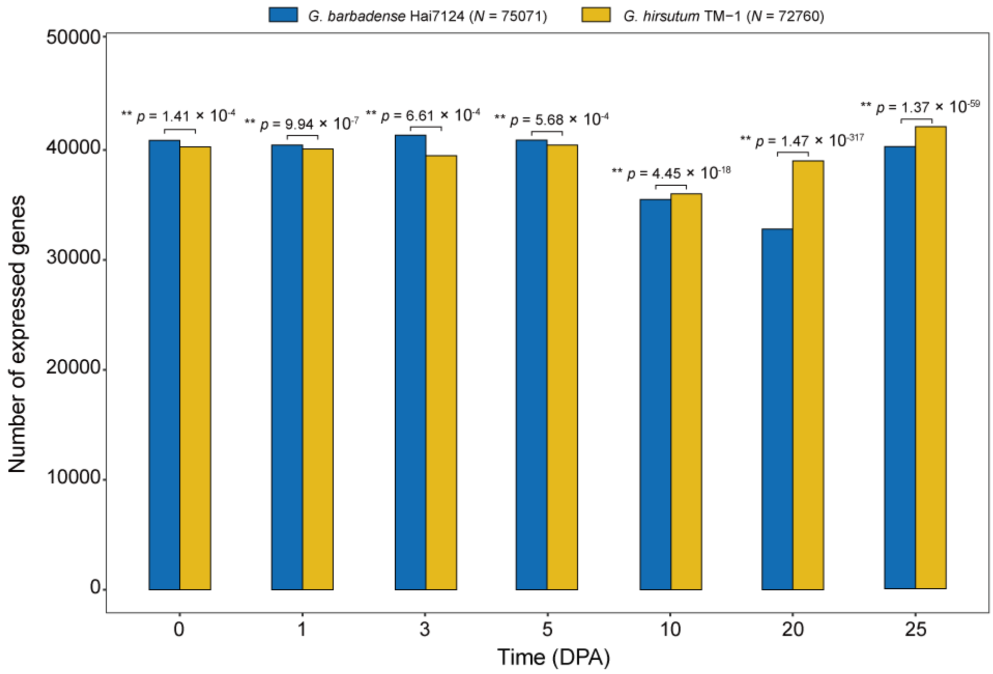

Figure S4. Numbers of differentially expressed genes in *G. hirsutum* (TM-1) and *G. barbadense* (Hai7124). Blue bar represents Hai7124. Yellow bar represents TM-1. **: $p < 0.01$, Fisher's exact test.

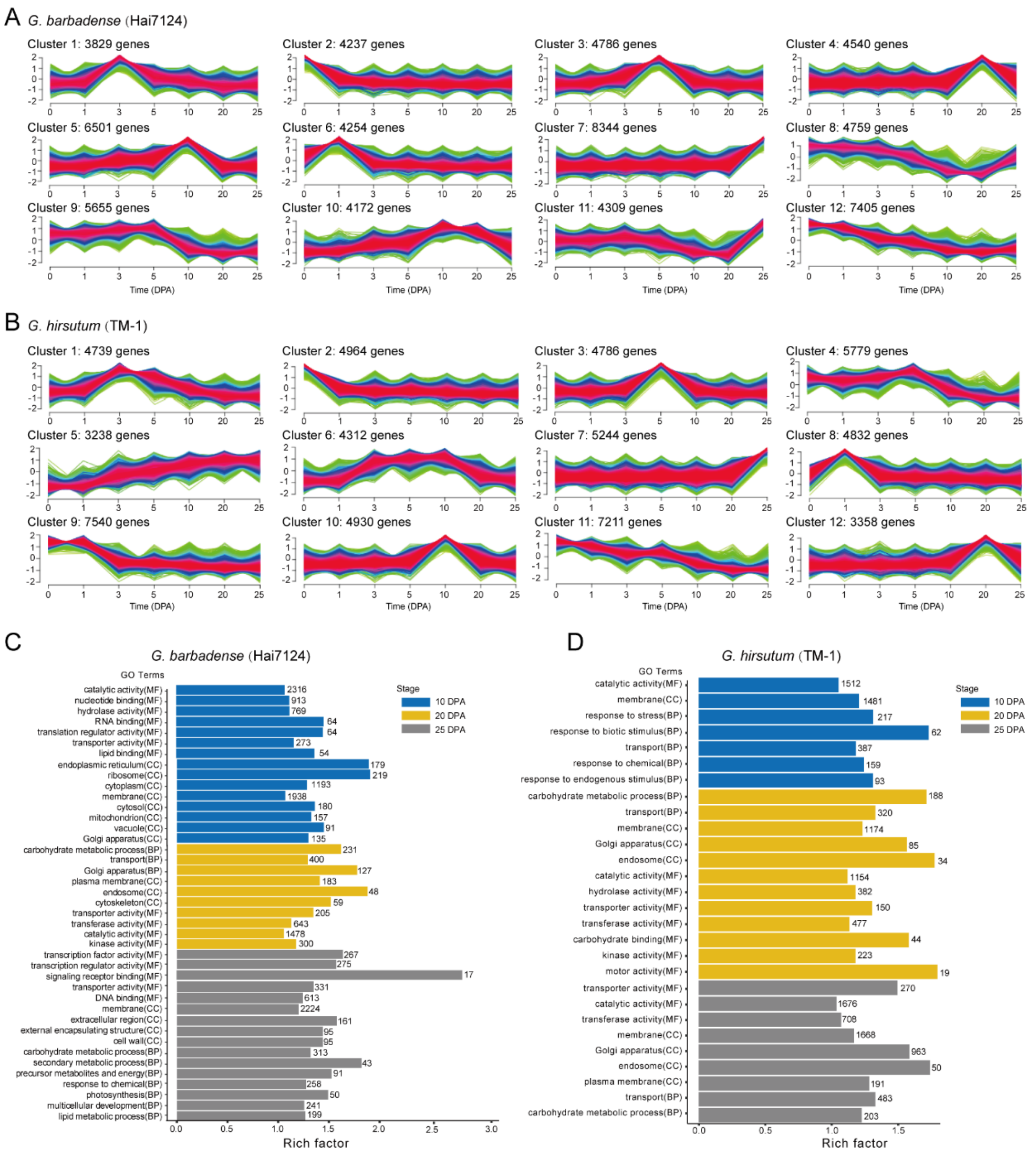

Figure S5. Clustering results for *G. barbadense* (Hai7124) and *G. hirsutum* (TM-1) with GO analysis. Cluster results of Hai7124 **(A)** and TM-1 **(B)**. GO analysis of genes in Hai7124 **(C)** and TM-1 **(D)**.

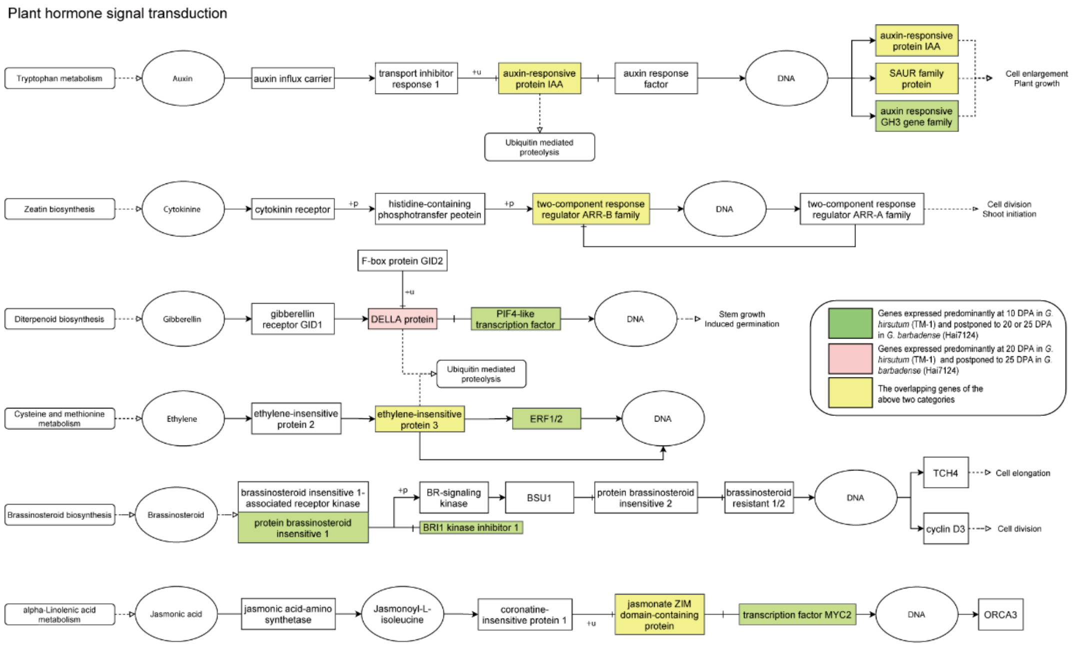

Figure S6. Diagram of postponed genes participated in plant hormone signaling pathway.
